# Can Fish Escape the Evolutionary Trap Induced by Microplastics?

**DOI:** 10.1021/acs.est.4c09932

**Published:** 2025-03-04

**Authors:** Weiwenhui Liang, Bowen Li, Amelia Munson, Qiqing Chen, Huahong Shi

**Affiliations:** 1 State Key Laboratory of Estuarine and Coastal Research, East China Normal University, Shanghai 200241, China; 2 State Environmental Protection Key Laboratory of Environmental Pollution Health Risk Assessment, Research Center of Emerging Contaminants, South China Institute of Environmental Sciences, Ministry of Ecology and Environment, Guangzhou 510655, China; 3 School of Biodiversity, One Health & Veterinary Medicine, University of Glasgow, Glasgow G12 8QQ, United Kingdom; 4 Department of Wildlife, Fish & Environmental Studies, Swedish University of Agricultural Sciences, Umeå 750 07, Sweden

**Keywords:** foraging, competition, ecological risk, multimodal cues, microplastics

## Abstract

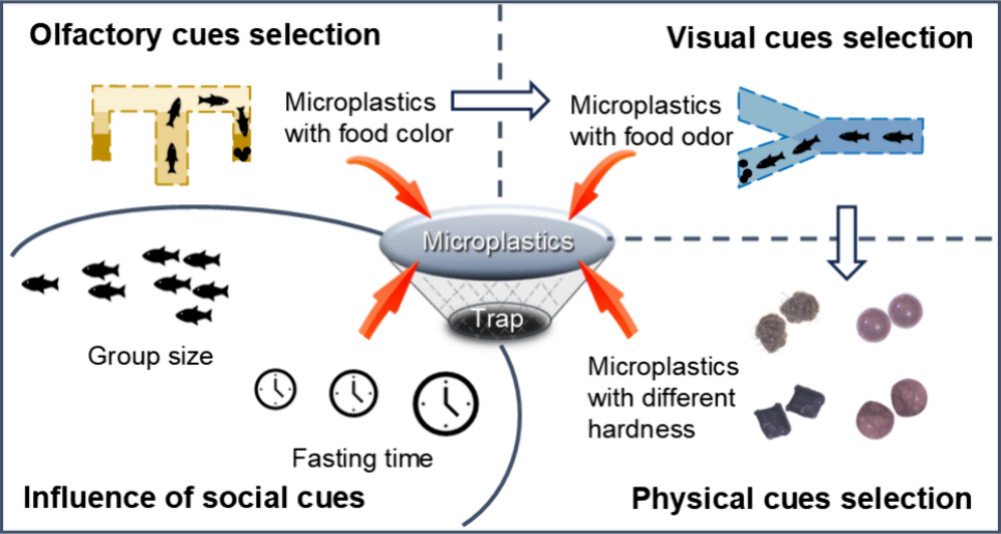

Microplastic (MP) ingestion acts as an evolutionary trap with various ecological consequences. Cues that lead animals to respond differently to MPs are key factors driving MP ingestion, yet they remain poorly understood. Here, we quantified the susceptibility of three fish species to different types of MPs across different social contexts. Our results showed that bass were more attracted to MPs that resembled food visually, whereas carp tended to select MPs that shared olfactory cues with food. Goldfish relied more on oral processing to make foraging decisions on MPs. Structural differences in the oropharynx supported these discriminated oral processes. Enlarged group size and fasting time altered the foraging behaviors of MPs of goldfish and bass, both of which were suction-feeding species. Such behavioral changes, regardless of whether fish ultimately ingested or rejected MPs, could pose indirect costs to fish. However, changed group sizes and fasting times did not affect the intake of MPs by the filter-feeding carp. We also proposed four pathways causing the MP-induced evolutionary trap and discussed the potential of fish to escape this trap. Our results contribute to experimental and theoretical understanding of the ecological risks posed by MPs to aquatic species.

## Abstract 2

From the detection of cues to swallow decisions for microplastics, fish’s behaviors were affected by cues of microplastics and social contexts of fish in a species-specific manner.

## Introduction

Human-induced rapid environmental change is widespread, prompting claims that we are now living in an epoch altered by the significant impact of human actions.^1^ As a result of human actions, animals need to increasingly make decisions based on new environmental cues.^2^ Microplastics (MPs) are tiny plastics that have been found in all types of ecosystems.^3,4^ Diverse species are reported to ingest MPs with variable abundance.^5,6^ Unlike most chemical pollutants that are dissolved in water, MP particles can lead animals to forage, capture, and swallow them actively. While MPs are ingested passively in some cases, their sensory characteristics may entice animals to forage on MPs actively despite negative fitness outcomes.^7,8^ The evolutionary trap is a situation in which animals respond in maladaptive ways to a changing environment, leading to negative consequences.^9^ If animals continue to behave in ways that increase their likelihood of ingestion of MPs, MP ingestion can be considered as an evolutionary trap.^10^

The specific cues associated with MPs that lead to ingestion are important in mitigating the trap. Unfortunately, while numerous studies have investigated the number and characteristics of MP particles being ingested and the consequences of ingestion, less research has focused on the specific cues of MPs that lead to animals’ foraging decisions.^11,12^ Central to this issue is whether a species or individual can distinguish the suite of cues (e.g., odor, color, size, and flavor) associated with MPs from historic prey items.^7^ Therefore, behavioral responses of animals to these cues associated with active selection are needed to estimate the selective feeding of MPs.^13^ It has previously been shown that a variety of species reject microplastics.^14−16^ Nevertheless, it is now well understood whether this rejection is related to the specific cues of the different MPs. A more nuanced understanding of specific cues and the likelihood of ingestion or rejection of MPs could help to target efforts to reduce the severity of the trap.

The contexts in which animals encounter MPs also may alter the likelihood of ingestion and, thus, the severity of the trap. For example, social cues are important factors that influence foraging decisions in many species.^17^ Group living benefits animals by expanding sensory sensitivity and increasing foraging efficiency, but it also increases competition for resources.^18,19^ If MPs are considered as resources and this inaccurate cue is exploited by individuals in groups, effective foraging may become useless or even harmful, and higher competitive pressure may mislead individuals into ingesting more MPs.^20^ Individuals also copy the foraging decisions of conspecifics, which may spread the trap through a social group.^21^ Moreover, individuals usually integrate external social cues with internal physiological states (e.g., hunger) to make feeding decisions.^22^ Competitors in poor conditions may be more likely to select less profitable items, or even MPs, because of greater competition.^23^ Most research on MPs though does not consider the social contexts of foraging, which means that we lack an understanding of the role of social facilitation in the MP-induced evolutionary trap.

To fill these gaps, we analyzed the species-specific fish foraging selection of MPs with or without similar cues to those of food. In addition, we investigated the effect of perceived competition on MP ingestion by testing fish in different group sizes and after different fasting times. Studying various cues used by fish during foraging can help us understand the motivation behind MP consumption with the aim of identifying species that are more susceptible to MPs in the evolutionary trap framework.

## Materials and Methods

### Fish Husbandry and Microplastic Preparation

Juvenile goldfish (*Carassius auratus*, Linnaeus, 1758), largemouth bass (*Micropterus salmoids*, Lacepède, 1802), and bighead carp (*Aristichthys nobilis*, Richardson, 1845) were selected as test species according to the different feeding strategies.^24^ Goldfish and bass use suction feeding to capture food. However, bass primarily targets larger prey, while goldfish have a more varied diet. Carp, on the other hand, are filter feeders, extracting food from the water. All three fish are widespread species, which respond to sensory cues and display social interactions within groups.^25−27^ Fish were acclimated to home tanks (40 × 25 × 20 cm) for 2 weeks. The average total length, weight, and mouth size of each species were measured (Table S1). Goldfish and bass were fed food pellets, while carp were fed food fragments (Qianfu Aquarium Products Co., Xiamen, China) twice daily. Fish rearing and handling procedures were approved by the ethical approval for animal experimentation of East China Normal University.

The shape and size of MPs were selected to match species-specific food preferences. MP pellets with an average size of 2.13 ± 0.09 mm were exposed for goldfish and bass, and MP fragments with an average size of 0.26 ± 0.09 mm were exposed for carp. Alanine was selected as the olfactory cue because it is a common feeding stimulant of fish feed and could be released from biofilms on MPs.^28,29^ Virgin MPs used for the olfactory cue test were soaked in an alanine solution (0.2% *v/v*) with shaking for 72 h. The certain odor cue on MPs was analyzed through high-performance liquid chromatography with the Agilent 1260 HPLC System (Agilent, USA, Figure S1). MPs with food colors (brown) and MPs with nonfood colors (a mix of blue, yellow, red, and green together) were selected as the visual cue. For the physical cue test, we used four floating MP polymers, distinguished by hardness and surface texture (Table S2). Hardness was measured using a sclerometer (LX-C-2, JB6148–92, China). The surface texture was examined by scanning electron microscopy (SEM, GeminiSEM 450, ZEISS, Germany). For the test of X-ray micro-CT, specially manufactured MP pellets containing 30% BaSO_4_ were selected as developers to observe the location of MPs in the fish oral cavity. Virgin MPs with food color were used in the group size and fasting time tests. In addition to the specific cues that were prepared for each test, the other cues of MPs remained the same across all tests. Additional details on fish acclimation condition and MP pretreatments are provided in the Supporting Information.

### Experimental Setup

Four tests were conducted to assess fish foraging responses to MPs with olfactory cues (alanine odor VS virgin), visual cues (food brown VS other colors), physical cues (different hardness and surface texture), and different group sizes and fasting times (Figure S1). Fish behaviors were recorded using a camera (acA2500–60uc, Basler, Germany, 25 fps) above different arenas under two LED shadowless strips (power: 8 w, luminous flux: 360 lm). Tests were performed on a vibration isolation optical table (OTP15–10, Zolix) with shade cloths to ensure minimal disturbance of the fish. Each test included a 10 min acclimation for fish and an 11 min MP exposure. MPs with different cues (0.8 items/L for pellets to goldfish and bass and 2 mg/L for fragments to carp) were added to arenas in all tests based on the pilot study except for the fasting time test. In the fasting time test, three fish were exposed to MPs at three times the concentration used in the pilot study (1 fish only) to focus on fasting effects under sufficient food conditions. Different behavioral parameters of fish were analyzed in different tests (Table 1). Due to the filter-feeding strategy, foraging behaviors could not be recorded for carp. Instead, carp were dissected to examine the MP intake in the gills and guts after group size and fasting time tests to assess the impact of competition. Each fish was tested only once per condition to avoid learning effects. The details of MP dose and exposure time selection are provided in the Supporting Information.

**Table 1:** Definition of Behavioral Parameters Used in Different Tests

**behavioral parameters**	**definition**
Olfactory and Visual Cue Tests	
first selection proportion (%)	the proportion of fish that select the cue arm for the first choice
first selection time (s)	the time between when fish swim freely after removing the partition and when fish first enter either cue arm
duration in the cue arms (s)	the cumulative time spent in each cue arm in the 3 min after the first selection
Physical Cue, Group Size, and Fasting Time Tests	
response time (s)	the time between the introduction of MP and individual capture of MPs
capture frequency (times)	total occurrences of fish capture MPs
retention time (s)	the time between fish capturing and spitting out MPs

#### Olfactory and Visual Cue Tests

**Figure 1:**
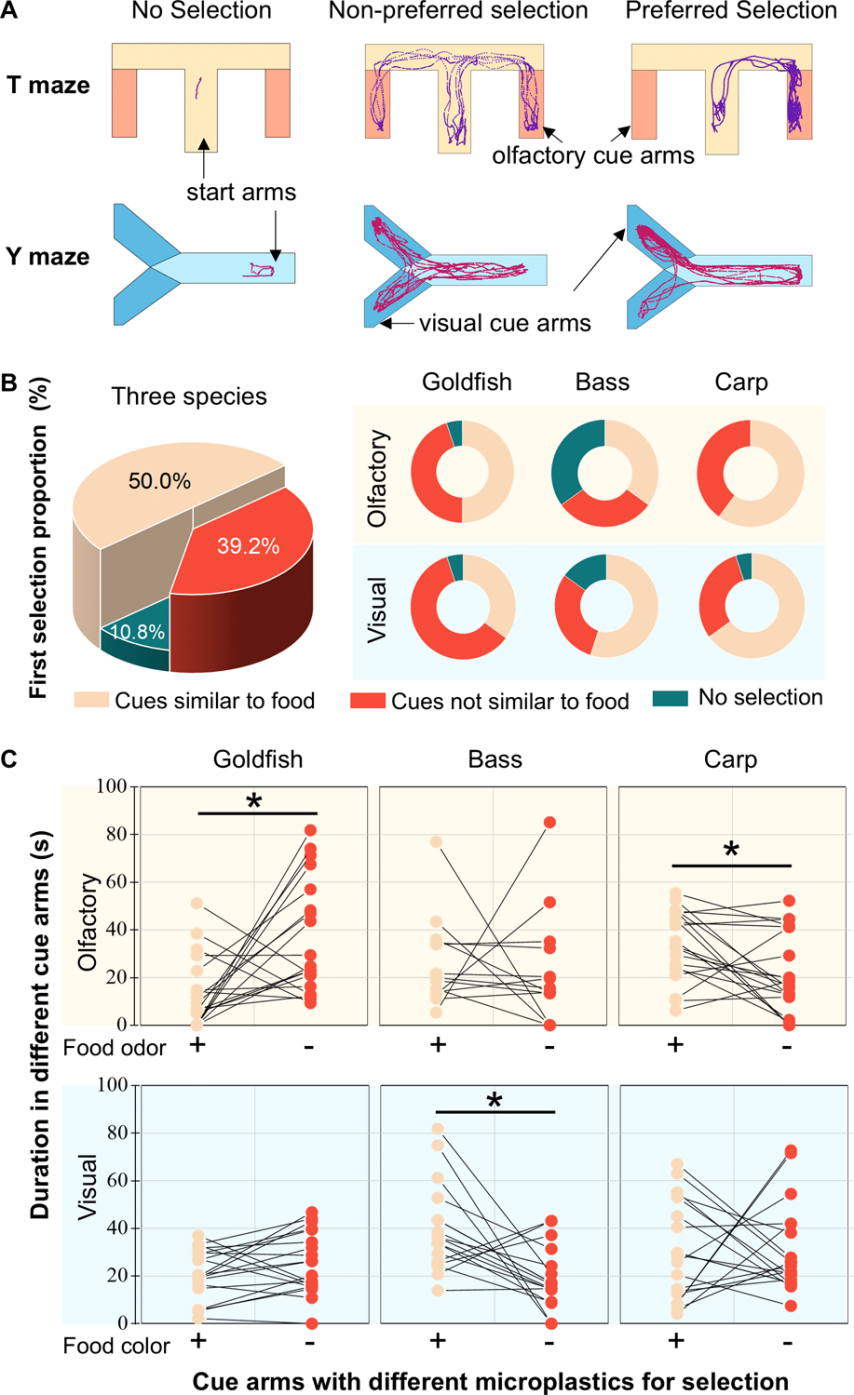
Selection of microplastics based on olfactory and visual cues. Sample trajectories for three types of responses in the “T” and “Y” mazes (A). Fish could enter neither arm during the whole trial (no selection), or select but exhibit either no difference in duration between cue arms (non-preferred selection), or select and exhibit longer duration in one of the cue arms (preferred selection). Proportion of all three species and proportion of each species that entered each cue arm for the first choice or did not select (B). Duration of fish in arms of MPs that had food odor/color (+) or had nonfood odor/color (−) during the first 3 min of the test (C). Only the first 3 min were analyzed for duration to avoid learning during the interaction time between fish and MPs with different cues. Only the fish that entered at least one cue arm were analyzed for the duration in the cue arms. * indicates the significant difference at the 0.05 level.

A “T” maze was used for olfactory cues comparison to reduce visual selection and a “Y” maze was used for visual cues comparison.^30^ Each maze was made of acrylic and consisted of a start-arm and cue arms (40 × 25 × 20 cm, Figure S1B,C). The floor and walls of the mazes were frosted white to decrease distraction from the environment. Twenty randomly selected individuals of each species were separately used for olfactory and visual cue tests. Each fish was transferred individually into the start arm and isolated behind a removable partition (Figure 1A). MPs with different cues were added at the far end of each cue arm after the fish was transferred to the maze. After 10 min of acclimation, the partition in the start arm was gently lifted, and the fish was allowed to explore the arena. Control tests in each maze were conducted with the selection of the cue arms with blank or food cues (Figure S2). The cue position was counterbalanced randomly on each side of the cue arms in selection tests. Trajectories of fish were analyzed using idtracker.ai.^31^

#### Physical Cue Test

Six goldfish and six bass were randomly selected from the home tank and exposed to MP pellets individually in round transparent acrylic tanks (30 cm in diameter and 20 cm in height). MPs were added in the central area of tanks after 10 min of fish acclimation.

#### Group Size and Fasting Time Tests

For the group size test, goldfish and bass in groups of 1, 2, and 5 conspecifics were replicated 5 times (i.e., a total of 5 individuals in group size 1, 10 individuals in size 2, and 25 individuals in size 5). For the fasting time test, groups of three individuals were fasted for 0.5, 24, 48, or 72 h and then exposed to MPs. Each fasting time had three replicates (*n* = 9 individuals per species). In addition, six specific foraging steps of goldfish and bass were diagnosed to understand the entire foraging process that contributes to the susceptibility to being trapped by microplastics (Table S3). The first 3 min of recordings of five individuals per species were manually labeled using Adobe Premiere Pro 2023.

### X-ray Micro-CT of MPs *In Situ*

Ten individuals of each species were randomly selected to be exposed to MPs with BaSO_4_. Goldfish and bass were subjected to slow-released anesthetization after observation of MP capture. Carp were anesthetized after 10 min of MP fragments exposure. All fish were gently transferred into 75% alcohol solution for 24 h after anesthetization. The positions of the MPs in the oral cavities of these fish samples were then observed using micro-CT (SkyScan 1272, BRUKER, Belgium). Additional details on slow-released anesthetization are provided in the Supporting Information.

### Statistical Analysis

In the olfactory, visual, and physical cue tests, the Mann–Whitney U test and the Kruskal–Wallis test were used to verify the differences in first selection time, duration in the cue arms, capture frequency, and retention time. In the group size and fasting time tests, three generalized linear mixed models (GLMM) were applied to the response time, capture frequency, and retention time of goldfish and bass. Each model included group size, fasting time, and fish species as fixed factors. The other GLMM model was applied to the intake of microplastics by carp. Group size, fasting time, and anatomical sites were included as fixed factors. Fish ID nested within the group was included as a random factor in the four models. A Poisson distribution was applied to the intake and capture frequency. A Gamma distribution was used for retention time and response time. Model outputs are in Table S4. All analysis was performed using R v. 4.4.0.^32^ The data were presented as mean ± standard deviation (SD).

## Results and Discussion

### Selection of Olfactory and Visual Cues of MPs during Foraging

The tested fish had different selection preferences for olfactory cues in a “T” maze and visual cues in a “Y” maze (Figure 1A). Among the 20 goldfish tested for visual cue preferences, only 35% selected food-colored MPs (brown, Figure 1B). Similarly, among the 20 goldfish tested for olfactory preferences, 50% selected MPs with food odors (alanine). Goldfish stayed in the cue arm with virgin MPs longer than the arm with MPs with food odor (Figure 1C, *p* < 0.05). Bass, on the other hand, selected MPs in food brown color more often (55%) and stayed in the cue arm with brown MPs longer than that with mixed color MPs (*p* < 0.05). There was no difference for bass in the first olfactory selection proportion and duration in the cue arms between MPs with food odor and virgin MPs (Figure 1 B,C). Bass also made their first selection much more quickly in the visual test than in the olfactory test (Figure S3*, p* < 0.05), which suggests that they may be more likely to make selections based on visual cues. For carp, 60% of tested individuals selected MPs with food odor and 65% of individuals selected MPs with food color as their first choice. However, unlike goldfish and bass that tended to stay in the same arm they chose, carp tended to move continuously around the mazes in both olfactory and visual tests. Therefore, the results of the first selection of carp were less informative. Carp also made their first selection in both olfactory and visual texts much faster than goldfish and bass (Figure S3, *p* < 0.05). When carp entered the cue arm with MPs that had food odor, they stopped swimming and spent a longer time in this arm than in the arm of virgin MPs with no food odor (*p* < 0.05).

Generally, olfactory and visual cues provide fish with information for locating and capturing food.^33^ It is believed that animals forage for MPs selectively because of the close resemblance of visual or chemical cues between MPs and foods.^10,34−37^ Our results, however, showed that there was no difference in the overall first selection proportion of 3 fish species between MPs that shared odor and visual cues with food and MPs that had less overlapped cues with food (*p* = 0.090). This may be due to the fish’s species-specific selections between olfactory and visual cues of MPs. Our results suggest that the color of the MPs could influence the foraging selection of bass to MPs. Bass is a food-specific species (specialists), while goldfish are generalists that feed on a variety of items and typically are willing to sample novel items.^25,38^ This could be the reason that goldfish were more likely to select MPs with nonfood colors and stayed in the cue arm of nonfood odor MPs longer than that of food odor MPs. Thus, goldfish may be more likely to fall into multiple MP-induced traps because they were less discriminatory between food and nonfood items compared to specialists. Ingestion of small MP fragments was supposed to be unavoidable for carp following the filtering feeding strategy. Although we could not record the foraging behavior of carp, the speed of them making decisions and time spent in an arm after making a choice suggest that carp relied more heavily on odor compared to visual cues.

### Physical Cues and Their Role in Ingestion or Rejection of MPs

**Figure 2:**
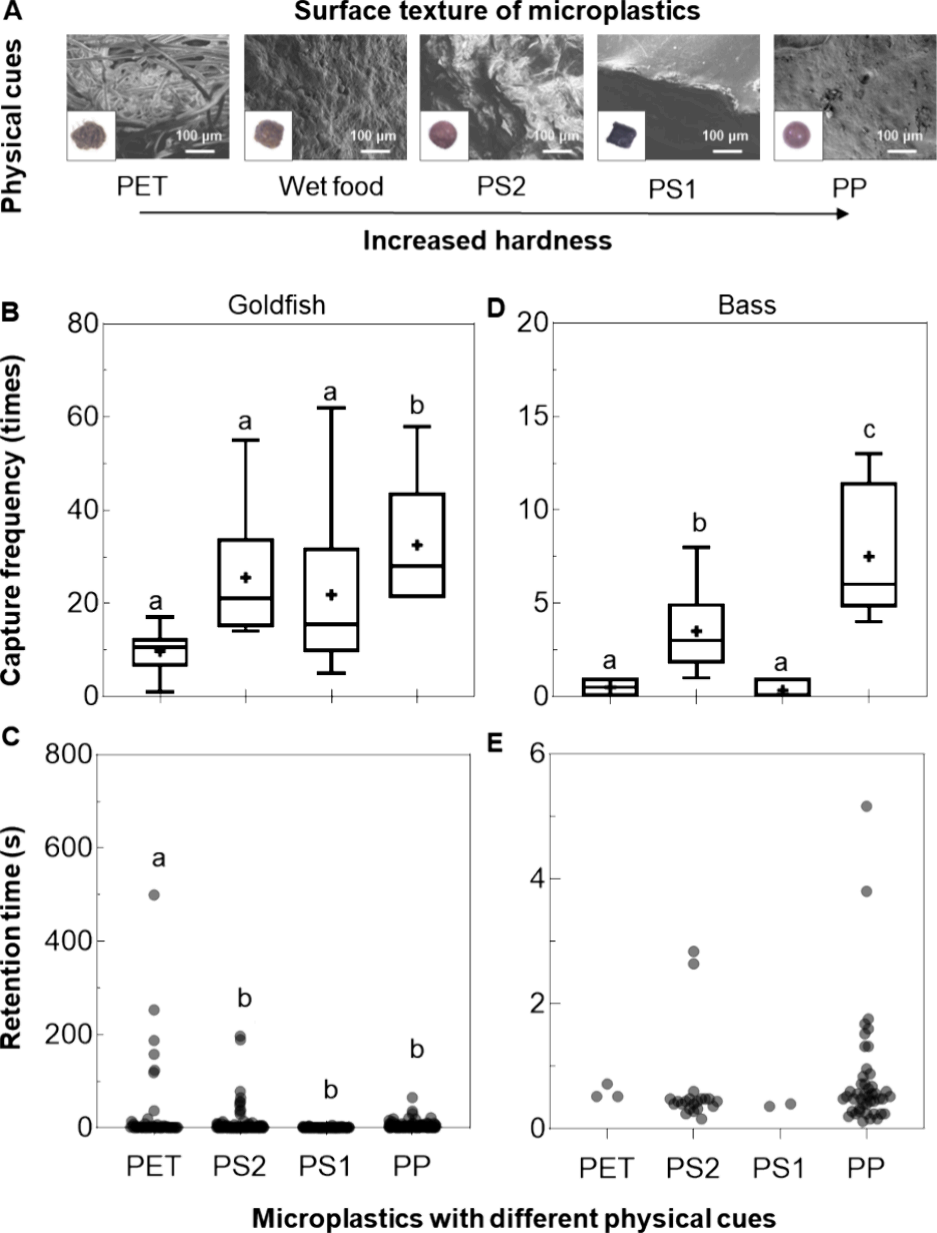
Different responses of goldfish and bass to microplastics with different physical cues. Hardness and surface texture of MPs and food (A). MP pellets in the bottom left corner of each SEM picture are of similar size, averaging 2.13 ± 0.09 mm. Capture frequency (B, D) and retention time (C, E) of different types of MPs were obtained for goldfish and bass. Note the different units of the *y*-axis for goldfish and bass on both measures. a, b, and c indicate the significant difference at the 0.05 level.

Goldfish and bass captured all four types of MPs repeatedly and kept pellets for varying lengths of time before spitting them out (defined as retention time). The softest pellets were polyethylene terephthalate (PET) with brushed surface texture, followed by lumpy polystyrene foam (PS2), sharp manufactured polystyrene (PS1), and smooth polypropylene (PP), respectively (Figure 2A). The hardness of PET pellets showed no difference from wet food. Although PP pellets were captured by goldfish significantly more frequently than other tested pellets (*p* < 0.05, Figure 2B), they were rejected quickly after each capture compared to the softer PET pellets (*p* < 0.05, Figure 2C). While we could not confirm the swallowing of MP pellets without invasive sampling, one out of six goldfish retained one PET pellet until the end of filming. The longer retention time of PET pellets compared to other MPs in the goldfish’s oral cavity suggests that goldfish were more attracted to softer MPs similar to foods’ hardness. Bass captured MPs less frequently and retained MPs for a shorter time compared to goldfish (*p* < 0.01, Figure 2B–E). Bass captured PET and PS1 pellets less frequently than PP and PS2, which were similar in surface texture to lumpy wet food (*p* < 0.05, Figure 2D).

Texture is a combination of different physical characteristics and can be used by fish to make decisions during capturing, swallowing, or rejecting objects.^39^ The most common hypothesis of MP rejection after capture is that the taste of MPs is different from real foods.^40,41^ However, MPs that taste differently from food are still ingested by many species.^10^ This phenomenon suggests that some other mechanisms may lead animals to MP ingestion. Physical cues of MPs, including hardness and surface texture, could be one of the mechanisms that aid in the oral decision-making process of swallowing MPs. Unlike visual or olfactory cues that can be judged from further distances, physical cues of MPs become more important after the fish have already captured them. Goldfish, notably, spent much longer time retaining all types of MPs in their mouth than bass, suggesting that goldfish may rely more on the oral-chemical and oral-physical sense to make foraging decisions for MPs.^42,43^

**Figure 3:**
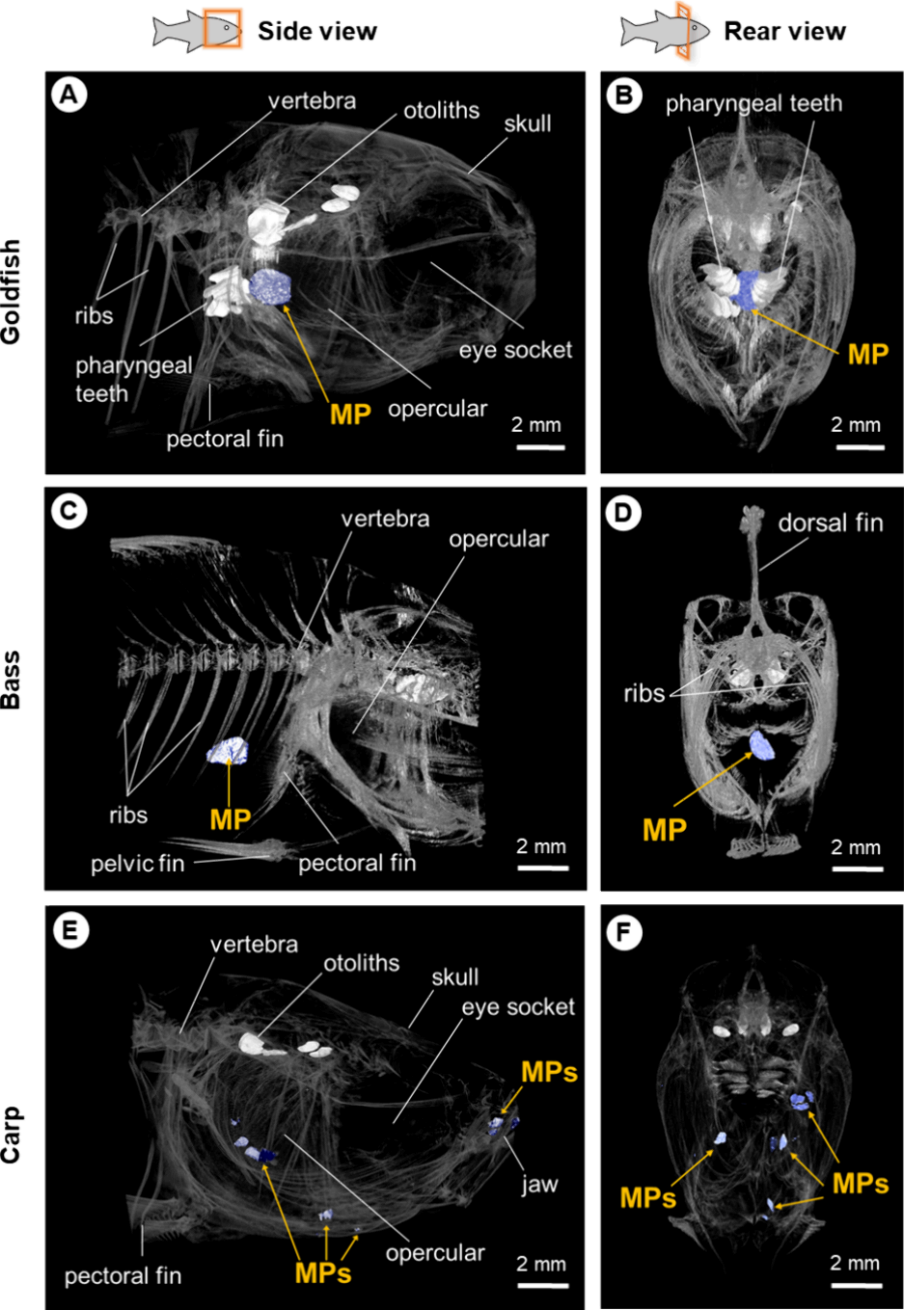
Oral structure of three fish species and location of microplastics inside them. Side and rear views of goldfish (A, B) and bass (C, D) with the MP pellet. Side and rear views of carp (E, F) with MP fragments. Images were obtained from the refined fish model by using microcomputed tomography (micro-CT). MPs with BaSO_4_ were shown as the same color as bone and then MPs were marked by pseudocolored mapping in blue color.

The X-ray micro-CT showed the location of MPs inside the fish after they had captured the MPs. MPs were found near the pharyngeal teeth in 60% of goldfish samples (Figure 3A,B). Pharyngeal teeth are located in the pharyngeal arch of cyprinids and are used to process foods.^44^ Goldfish have noticeable pharyngeal teeth in the micro-CT images. The longer retention time of MPs by goldfish than by bass may be because they were trying to grind MPs with pharyngeal teeth. It was difficult to observe the position of MPs in bass because they rapidly spit MPs out before they were anesthetized. Only one MP pellet was observed in a bass (Figure 3C,D). MP fragments were found in all carp samples on either side of the gills (Figure 3E,F).

These structural pathways, including the grinding by pharyngeal teeth and gill flushing, aid fish in rejecting MPs and escaping the evolutionary trap.^24,45^ However, the different oral structures of fish with different feeding strategies were related to the types of MPs that were likely to trap fish. For example, filtering carp was more likely to ingest small-size MP fragments rather than MP pellets. Even if the fish do not ultimately ingest MPs, these MPs still could be a trap. MPs larger than interfilament space were more likely to be blocked on the gills of carp, which also may cause mechanical injuries on the gills.^46^ If goldfish keep investing more time in the oral handling process to decide whether to reject MPs or not, this would reduce their time spent on true food foraging and lead to indirect costs. It is hard for now to find a one-size-fits-all solution that could reduce all kinds of MP occurrences in the environment.^10^ It will also be hard to identify a key cue of MPs that is not attractive to any fish if different species exhibit different feeding preferences for MPs. Therefore, it is more important to study the species-specific foraging ecology of MPs because it will help us identify the key species that are more susceptible to MPs.

### Influence of Group Size and Fasting Time on MP Ingestion

**Figure 4:**
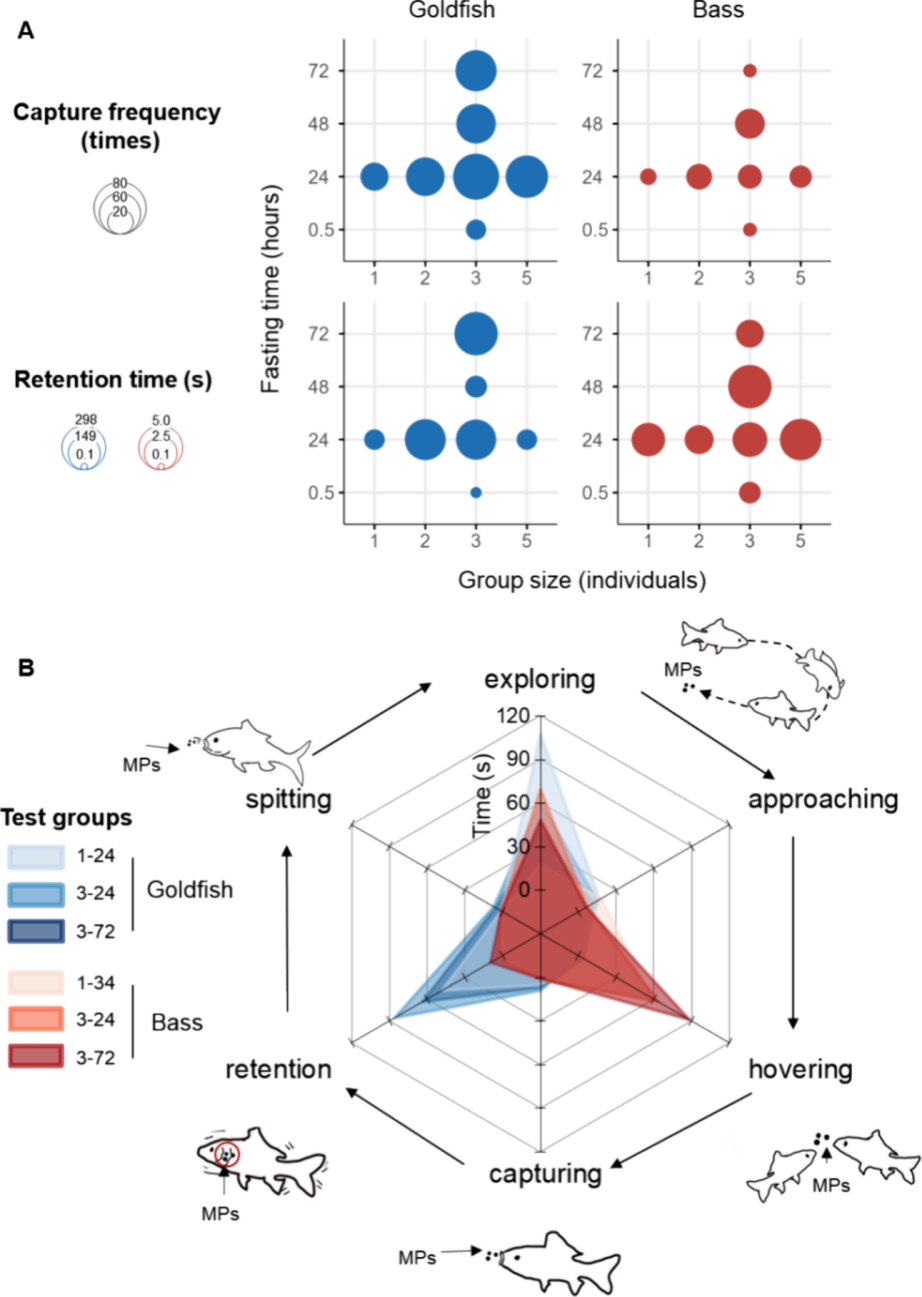
Influence of different group sizes and fasting times on the foraging behavior of fish to microplastics. Capture frequency and retention time of MPs in goldfish and bass in different group sizes or following different fasting times (A). Average time engaged in different feeding behaviors of fish in different group compositions in the first 3 min recording (B). Foraging was diagnosed into six steps: random swimming (exploring), turning and swimming toward MPs (approaching), pausing near MPs (hovering), capturing MPs following holding in the mouth (retention), and ejecting MPs from the mouth (spitting). Group 1-24: 1 individual, fasting 24 h; Group 3-24: 3 individuals, fasting 24 h; Group 3-72: 3 individuals, fasting 72 h.

Goldfish and bass retained MPs longer (*p* < 0.05) and had an increased trend to capture MPs in larger groups (Figure 4A), which suggests that fish were willing to investigate MPs as possible food items for longer to avoid an immediate loss to competitors. However, goldfish and bass retained MPs shorter following longer fasting times (*p* < 0.001, Figure 4A). Response time and capture frequency of MPs by goldfish and bass were not influenced by fish group sizes or by the following fasting times. There was also no difference in the intake of MPs by carp in different group sizes or following different fasting times (Figure S4). However, the frequency distributions of fish capture behavior were different in different group sizes (*p* < 0.05, Figure S5B), which indicates the changed foraging pattern of fish due to social cues involved.

To expand foraging change from one behavior to the whole foraging process, we diagnosed foraging for MPs into six main steps (Figure 4B). Goldfish typically spent more time retaining MPs in their mouth compared to other steps in the foraging process (*p* < 0.05, the blue radars in Figure 4B). Bass spent more time hovering near the MPs compared to other steps (*p* < 0.05, the red radars in Figure 4B). Throughout the group size and fasting time tests, both species spent less time retaining MPs and captured them less frequently (*p* < 0.05, Figure S5). Further details of the behavior patterns of the three species are summarized in the Supporting Information.

Social contexts, including competition and hunger level, can change the way that fish view cues in their environment.^47^ This point was evidenced by the changed foraging patterns of the fish in our study. Group size may influence perceived competition by increasing the population density and decreasing resource availability for each individual.^48^ Thus, fish may be more motivated to feed in larger groups.^49,50^ In our study, increased retention time and longer duration of capture behavior in larger groups supported the idea that fish were attracted to low-fitness MPs longer when they were in larger competition intensity. Food deprivation may make fish less discerning and more likely to ingest MPs if the immediate need for food is greater than the risk of making a mistake.^51^ However, fish did not capture MPs more frequently and retained MPs shorter following longer fasting times. This may be because fish have some information from the first couple of captures and are less likely to invest time in useless items when they are more in need of food.^52^ Longer fasting periods also can influence the locomotor behavior of fish associated with food searching and exploration.^53^ Locomotor behaviors in feeding traits of fish should be explored more in the potential for MP ingestion, especially for species like carp, whose foraging behaviors are hard to quantify. In addition, decreased capture occurrence and retention time during the tests suggest that fish can learn relatively rapidly that MPs are not food items. However, additional testing is required to determine whether fish retain this knowledge or must continue to capture MPs in more complex environments with other food items.

### Can Fish Escape the Evolutionary Trap Induced by MPs?

**Figure 5:**
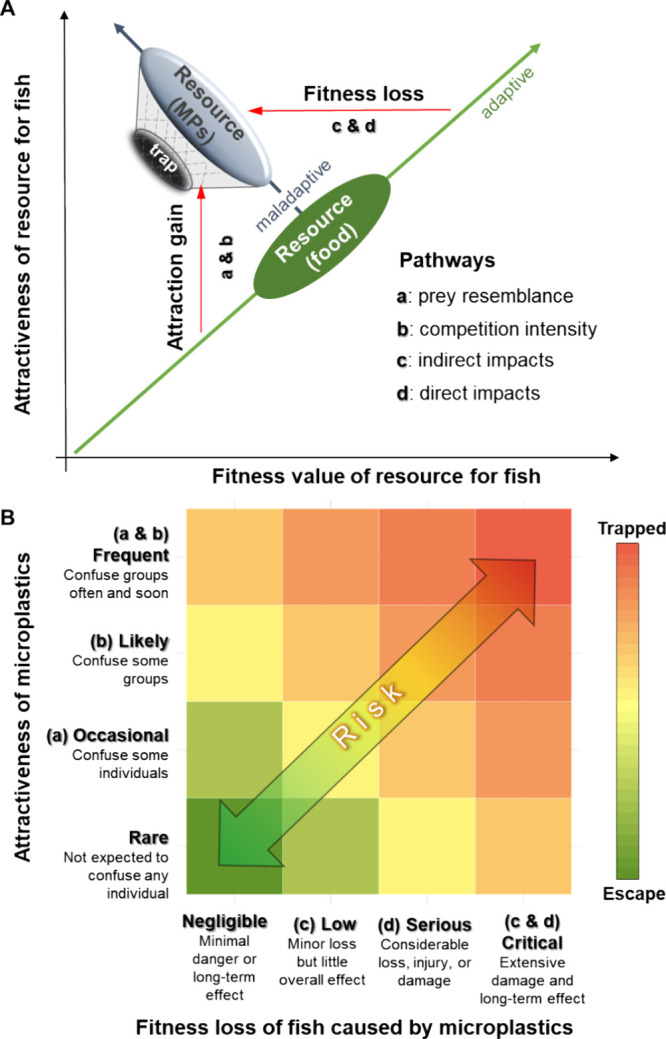
Microplastic-induced evolutionary trap for fish and the possibility to escape. Graphical representation of the possible pathways of the MP-induced evolutionary trap (A). This framework followed the general mechanisms by which animals fall for evolutionary traps posed by Robertson et al.^59^ The green arrow across the food resource represents an expected set of adaptive preferences: food items that have a higher fitness value should be more attractive to fish. The black arrow across the microplastics represents a maladaptive selection if fish prefer MPs or are unable to distinguish between MPs and food in different contexts. The risk of the MP-induced evolutionary trap and the possibility of escape are examined (B). Attractiveness of MPs and fitness loss of fish were measured by four discrete categories assumed according to trapping pathways a-d. The statements about these categories followed the suggestion of category scales in the risk matrix.^60^

The active decision-making process makes studying MPs’ risks from an evolutionary trap framework, particularly useful because there are multiple steps at which fish can either misuse cues or alter behavior to reduce negative fitness outcomes. We proposed a specific framework of the MP-induced evolutionary trap by accounting for four different pathways and risks of falling into this trap (Figure 5A). MPs can become an evolutionary trap through attraction gain of MPs and/or fitness loss of fish. MPs that have more overlapping cues with historic prey are more likely to be captured by animals (pathway a).^16,54,55^ Previous studies have shown that species' sensory thresholds for detecting cues can affect MP ingestion.^10,56^ In our results, the fish’s feeding behaviors were changed according to the cues of MPs from the detection (vision and olfaction) to swallowing (physical mechanoreception). Changed foraging patterns in different group sizes or following different fasting times also suggest that changes in social contexts could also affect the attractiveness of MPs to fish (pathway b). In terms of pathways that cause fitness loss of fish (c and d, Figure 5), it is important to consider both direct and indirect costs. In addition to the direct harmful effects (physical harm, oxidative stress, etc.), it is becoming increasingly clear that changes in fish feeding behavior or other energy traits pose indirect costs that must be considered to understand variation in fitness.^57^ It is crucial to consider the change in feeding performance of fish in the presence of MPs because fish will have additional energy costs by spending more time foraging MPs without finding nutritive food. This indirect impact of MPs may affect the overall foraging efficiency and future prey selection.^58^

In this framework, the risk of MP-induced evolutionary trap could decrease through reduction of the attraction of MPs and/or by mitigating the adverse impacts of MPs (Figure 5B). If fish select MPs actively (e.g., goldfish and bass), individual sensory feeding ecology and group social dynamics (pathways a and b) should be considered as key factors in determining the attractiveness of MPs as food. If there are costs for fish to MP ingestion, no matter direct or indirect, MPs that are more attractive to fish will pose a higher risk. This attractiveness may align with sensory feeding preferences or arise in social settings where groups exhibit heightened foraging eagerness. The latter scenario is more complex as multiple environmental conditions can influence the transfer of social information among group members.^61^ It may be inevitable for fish to ingest MPs passively (e.g., carp) due to the widespread distribution of small MPs in the environment.^62^ In this case, the severity of the trap can be assessed according to the extent and prevalence of MP ingestion impacts (pathways c and d). However, the attraction of MPs and their impacts can also interact simultaneously. Greater fitness costs might cause higher selective pressure against ingesting MPs, increasing the likelihood of escaping.^63^ Overall, if MPs become frequently attractive to fish while causing more critical impacts, then it will be more difficult to escape the evolutionary trap of MP ingestion.

## Environmental Implication

It is particularly crucial to understand the factors driving fish to actively forage for MPs because these factors may affect the MP-induced evolutionary trap severity in ways that passive ingestion cannot. However, the impact of active ingestion has largely been overlooked in the current MP risk assessment because fish typically reject MPs, preventing the significant accumulation of MPs in their bodies. In this study, we have important findings by exploring the factors that influenced the foraging behaviors of fish for MPs with environmentally acceptable concentrations. The attractiveness of MPs for fish was greatly influenced by the multiple cues of MPs sharing with food in a species-specific manner. Social contexts also played a role in changing the motivation of fish to forage for MPs. These findings provide new insights into the environmental risk of active MP ingestion at sublethal levels from individual feeding selection to group optimal decision-making across species and highlight the associated opportunity costs during foraging for MPs.

## Supporting Information Available

Supporting methods of the details of fish husbandry conditions, microplastic cues preparation, pilot study, two control tests for selection mazes, analysis of alanine, and X-ray micro-CT; Supporting results of further details of the influence of group size and fasting time on microplastic ingestion of carp, behavior patterns of three fish species to microplastics, and learning effects of fish to microplastics during group tests; five supporting figures of test setting and materials (Figure S1), control test of maze tests (Figure S2), first selection time of fish (Figure S3), intake of microplastics by carp (Figure S4), and behavior pattern of goldfish and bass (Figure S5); five supporting tables of the average fish length, weight, and mouth size information (Table S1), hardness value and surface texture of test materials (Table S2), definition of foraging steps (Table S3), and results of generalized linear mixed models (Table S4) (PDF)
