# Supplementary material for: Can Fish Escape the Evolutionary Trap Induced by Microplastics?
Source: Environ Sci Technol. 2025 Mar 4;59(10):4788–96. doi: 10.1021/acs.est.4c09932 (PMC11924217; doi:10.1021/acs.est.4c09932)
Supplement: Supplementary file 1 — es4c09932_si_001.pdf [file es4c09932_si_001.pdf]

# Supporting Information for

# Can fish escape the evolutionary trap induced by microplastics?

*Weiwenhui Liang[1, 3], Bowen Li[2], Amelia Munson[4], Qiqing Chen[1], Huahong Shi[1 *]*

[1] State Key Laboratory of Estuarine and Coastal Research, East China Normal University, Shanghai, 200241, China

[2] State Environmental Protection Key Laboratory of Environmental Pollution Health Risk Assessment, Research Center of Emerging Contaminants, South China Institute of Environmental Sciences, Ministry of Ecology and Environment, Guangzhou 510655, China

[3] School of Biodiversity, One Health & Veterinary Medicine, University of Glasgow, Glasgow G12 8QQ, United Kingdom

[4] Department of Wildlife, Fish & Environmental Studies, Swedish University of Agricultural Sciences, Umeå 750 07, Sweden

* Correspondence to: State Key Laboratory of Estuarine and Coastal Research, East China Normal University, Shanghai, 200241, China; e-mail: hhshi@des.ecnu.edu.cn; phone: 86(21)-62455593.

**This Supporting Information includes:**

Supporting methods: page S1-S4;
Supporting results: page S5-S9;
Figures S1 to S5: page S10-S14;
Table S1to S4: page S15-S18;

# Supporting methods

## 1. Fish husbandry condition

Juvenile fish individuals (4-6 months) were purchased from a local commercial supplier (Hongrui Ecological Agriculture Ltd, Zhejiang, China) and transferred to the laboratory in home tanks to acclimate for 2 weeks. Each tank was filled with dechlorinated water with an aeration system (4 L/min) at a stable temperature (22 ± 2 □) and pH (8.0 ± 0.2).

## 2. Microplastic cues preparation details

All microplastic (MP) pellets used in this study were manufactured and purchased from a raw material production plant (Kangjin Chemistry Company, Quanzhou, China) except for the polyethylene terephthalate (PET) fiber pellets which were handmade by cutting a long fiber. Five types of MP pellets were used in total (Fig. S1): PET, polystyrene foam (PS2), manufactured polystyrene pellet (PS1), polypropylene (PP) and PP pellets with 30% $BaSO_4$. Polyethylene (PE) fragments in different colors and PP fragments with $BaSO_4$ were ground from large plastic pellets using a ball mill (800C, LINGSUM, China). Before being exposed to fish, all virgin MPs were soaked in 75% ethyl alcohol solution for 24 h and then washed under flowing water. Polymer composition was identified with a micro-Fourier transform infrared spectroscopy microscope (Nicolet iN 10, Thermo Fisher, USA). All MPs used in this study were without alanine odor except the odor-prepared MPs in olfactory cues test (Fig. S1). All MPs used in this study were in color that was similar to food except the colored MPs in visual cues test.

## 3. The pilot study for exposure dose determining

To determine the exposure dose of MP pellets, 10 individuals of each species were tested in the round tanks. Individuals were filmed and fed with food pellets after 10 mins of acclimation. The number of food pellets captured by each fish was counted in 10 min. The average number of food pellets captured by goldfish and bass in 10 min was 7.28 ± 1.55 items/fish. Therefore, the exposure dose of MP pellets was 8 items/fish (0.8 items/L water). The exposure dose of MP fragments was the same as the weight of fed food fragments, i.e., 0.02 g/fish (0.002 g/L water). To compare food and microplastics in the selection maze, we also expose fish for 10 min to select different MPs with different cues.

## 4. Control tests for olfactory and visual mazes

Ten different individuals of each species were tested in a negative control group (blank in both cue arms) and a positive control group (food in one of the cue arms) to verify that fish were actively selecting items (a total of 120 individuals). There was no difference in the fish selection between the right arm and the left arm in the negative control group ($p > 0.05$, the Mann-Whitney U test). The fish selection proportion of food was significantly higher than the selection proportion of the blank arm ($p < 0.05$, the Mann-Whitney U test). These results showed that fish had preferred choices in both “T” and “Y” mazes, confirming the effectiveness of our experimental design. For visual and olfactory tests of different MPs, we found that fish kept capturing MPs actively in the first 3 min of each test.

## 5. Analysis of alanine by HPLC

The high-performance liquid chromatography (HPLC) analysis was conducted on the Agilent 1260 HPLC System (Agilent, America). HPLC separation was realized on a ZORBAX Eclipse XDB-C18 (4.6 x 250 mm, 5 μm) column. The mobile phases consisted of 0.1% formic acid in water (A) and acetonitrile (B). The flow rate was 1.0 mL/min and the column oven was maintained at 35 □. Standard L-alanine and Milli Q water were also tested to determine the retention time ($t_R$) of alanine.

To ensure that (1) microplastics contain the alanine odor without other chemical odors released in the water in the olfactory cue test, (2) microplastic pellets used in physical cue test have no difference in taste and olfactory information, we examined the chemical signal in (1) the dechlorinated water, (2) the water with different types virgin microplastics, (3) the water with microplastics after odor preparation. In addition, to ensure that alanine odor worked in the T maze during the olfactory selection statistics, we also examined the water in the cue arm with odor-prepared microplastics and with virgin microplastics at 0 min and 3 min, respectively. All water samples were concentrated $2\times10^4$ times before being injected for detection.

The peak corresponding to standard alanine is observed at $t_R$ = 2.35 min. The chromatogram of detected water samples showed that alanine was not detected in the dechlorinated water and the water with virgin microplastics. In the water with microplastic after alanine odor preparation, alanine is observed at $t_R$ = 2.38 min (Fig. S1). At the beginning of selection (0 min), alanine was only observed in the target cue arm that included odor-prepared MPs. After 3 min, Alanine was also found in the other cue arm. Compared to the alanine concentration in the target cue arm at 0 min, alanine

in the other cue arms after 3 min was at a very low level. We did not find any distinct peak in the liquid chromatography profiles of four polymers used in the physical cue test, which means the different polymers do not release olfactory and taste information.

## 6. X-ray micro-CT of MPs *in situ*

As soon as the MP capture of goldfish and bass was observed, anesthetic (low-concentration benzocaine, 10 mg/L) was slow-released to the round tank using an intravenous syringe (250 ml, 0.55×20 mm). Five equally spaced syringes were opened at the same time until the fish was observed to be anesthetized. The slow-release anesthetic was used to prevent individuals from spitting MPs under the stress of sampling. Since no MP ingestion was observed in goldfish and bass in the physical cue test, the impact of anesthesia on the likelihood of ingestion is minimal.

# Supporting results

## 1. The influence of group size and fasting time on microplastic ingestion of carp

No obvious foraging behavior of carp to MP fragments was observed in different group sizes and fasting times. Through digestion examination, MPs were found in the gills and guts of carp. Carp ingested MPs for an average of 13.0 ± 50.0 items/fish in the fasting test which was much more than that in the group size test (with an average of 1.4 items/fish, $p < 0.05$). There was no significant difference in MP occurrence in the gut and gill of carp in both group size test and fasting time test ($p > 0.05$, Fig. S4). Group size and fasting time may have little influence on carp's ingestion of MP fragments. The difference in the intake of MP fragments between the group size test and the fasting time test was related to the exposure concentration, which suggests the pollution level of MPs is the main factor for MP ingestion of filtering carp.

## 2. Behavior patterns of three fish species

Uptake and ingestion are seen as the main pathways for organisms to be affected by MPs at several levels of the aquatic system, including active and passive uptake[1, 2]. Active uptake refers to confusion of fish between food and MPs, which means fish select cues to discriminate MPs from food[3]. Passive uptake includes accidental ingestion during drinking or feeding and transfer of MPs with the food chain[1]. Through this pathway, fish may ingest MPs unintentionally[4]. Goldfish and bass had active behavioral responses to MPs in our study. Carp, as a filtering forager, may ingest MPs more passively.

### 2.1 Active foraging of goldfish and bass to MPs

For goldfish and bass who ingest MPs more actively, when they explored the environment and encountered MPs, they quickly approached and captured. After a period of retention in their mouths, they spit the MPs out or swallow them. The "capturing-retention-spitting-recapturing" pattern showed repeatedly while goldfish and bass foraged for MPs (Fig. 4B). MPs were retained in the oral cavity in both goldfish and bass for a longer period in the initial feeding stage and then both capturing and spitting occurred frequently and closely spaced (Fig. S5A, C). After making a few frequent captures, capture frequency was reduced for bass and they hovered for a longer time before capturing in groups 1-24, 3-24, and 3-72. Interestingly, capture of MPs occurred more frequently when aggressive behaviors occurred in the groups (like chasing and snatching, shown as the grey line in Fig. S5A) than when fish swam individually.

The distribution of capture behavior showed that goldfish and bass captured MPs for multiple rounds in larger groups (the area covered with shadow color in Fig. S5B). Larger group size motivated both goldfish and bass to maintain active capture of MPs for a longer time ($p < 0.05$, Fig. S5B). In the group size and fasting time tests, we recorded 1642 times of captures of MPs in goldfish (a total of 76 individuals). The retention time of MPs in the oral cavity of goldfish decreased with repeated captures (Fig. S5C). The total numbers of capturing of bass were 249 captures (76 individuals), which was much less than that of goldfish ($p < 0.05$, Fig. S5).

**2.2 Passive ingestion of carp to MPs**

The behavior response of carp to MP fragments followed the pattern of

"swimming\ventilating-ingesting -coughing", which was similar to that in a previous study with the intake of small-sized fibers[5]. Competition from changed group sizes and fasting times had little influence on MP ingestion of carp (Table S4). However, our result also showed that carp may have olfactory selection (Fig. 1), which suggests the possibility of selective feeding of filtering carp cannot be ruled out. Coughing behavior of carp was observed as a rejection pathway of MP fragments, which may be related to an instinct-rejected mechanism of fish to remove foreign objects through the gill[6]. Compared to active spitting of goldfish and bass, coughing of carp did not show changes over exposure time. Other foraging behaviors, such as exploring, approaching, and capturing, did not be observed.

## 3. Glimpse of learning from behavior patterns

Our results showed that fish individuals responded repeatably in approaching and capturing MPs instead of avoiding or ignoring MPs after they had already spit MPs out. Based on the signal detection theory, this phenomenon suggests that fish individuals behave in ways that increase their tries and ingestion of microplastics, which is maladaptive[7]. Our results also showed that fish (both individuals and groups) captured MPs more times in the first stage after encountering MPs and then decreased with time (Fig. S5). The decreases in capture frequency and retention time indicate that fish may learn something that contributes to rejecting MPs from former capturing. Learning is an important factor that gives animals flexibility to respond to changes in the environment[8]. If similar cues of MPs continue showing up, fish may give up the captures of MPs. However, the result that goldfish and bass kept capturing MPs for a

longer time in larger groups suggests that social information may change the way that fish estimate the risk of capturing MPs from learned information. Foraging in group contexts may exacerbate the occurrence of MP captures if other individuals copy the foraging decisions or ingest MPs more discriminately due to the higher competition perceived from group members. In this context, social learning may also be a way for fish to fall into MP-induced trap in some contexts. Learning process when fish make forage decisions is the key to estimating the likelihood of escaping from the MPs-induced evolutionary trap in the long term and at the group level.

## References


1. Roch, S.; Friedrich, C.; Brinker, A., Uptake routes of microplastics in fishes: practical and theoretical approaches to test existing theories. *Sci Rep* **2020,** *10* (1), 3896.
2. Jovanovic, B., Ingestion of microplastics by fish and its potential consequences from a physical perspective. *Integr Environ Assess Manag* **2017,** *13* (3), 510-515.
3. Savoca, M., The ecology of an olfactory trap. *Science* **2018,** *362* (6417), 904.
4. Li, B.; Liang, W.; Liu, Q. X.; Fu, S.; Ma, C.; Chen, Q.; Su, L.; Craig, N. J.; Shi, H., Fish ingest microplastics unintentionally. *Environ Sci Technol* **2021,** *55* (15), 10471-10479.
5. Liang, W.; Li, B.; Jong, M. C.; Ma, C.; Zuo, C.; Chen, Q.; Shi, H., Process-oriented impacts of microplastic fibers on behavior and histology of fish. *J Hazard Mater* **2023,** *448*, 130856.
6. Carlson, R. W.; Drummond, R. A., Fish cough response—a method for evaluating quality of treated complex effluents. *Water Res* **1978,** *12* (1), 1-6.
7. Trimmer, P. C.; Ehlman, S. M.; Sih, A., Predicting behavioural responses to novel organisms: state-dependent detection theory. *Proc Biol Sci* **2017,** *284*, 1847.
8. Dukas, R., Cognitive innovations and the evolutionary biology of expertise. *Philos Trans R Soc Lond B Biol Sci* **2017,** *372*, 1735.

## Supporting Figures

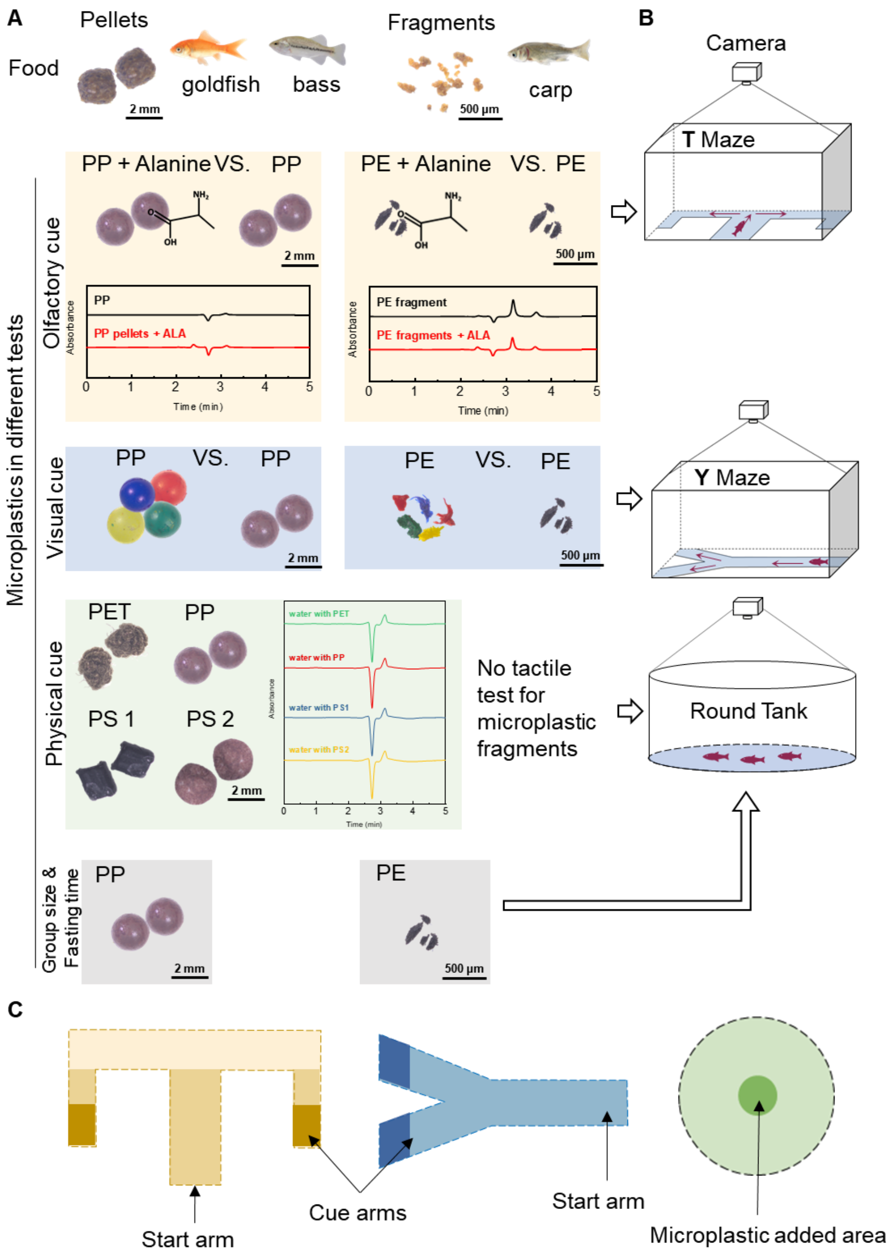


**Fig. S1. Microplastics (A) and test arenas (B-C) in different tests.**

Goldfish and bass were exposed to microplastic pellets and carp were exposed to microplastic fragments in all the tests. Chromatograms of odor-prepared PP pellets, PE fragments, and 4 types of virgin pellets were provided in **A**. The retention time of alanine is $t_R$ = 2.38 min. PP: polypropylene; PET: polyethylene terephthalate; PS1: polystyrene pellet; PS2: polystyrene foam; PE: polyethylene.

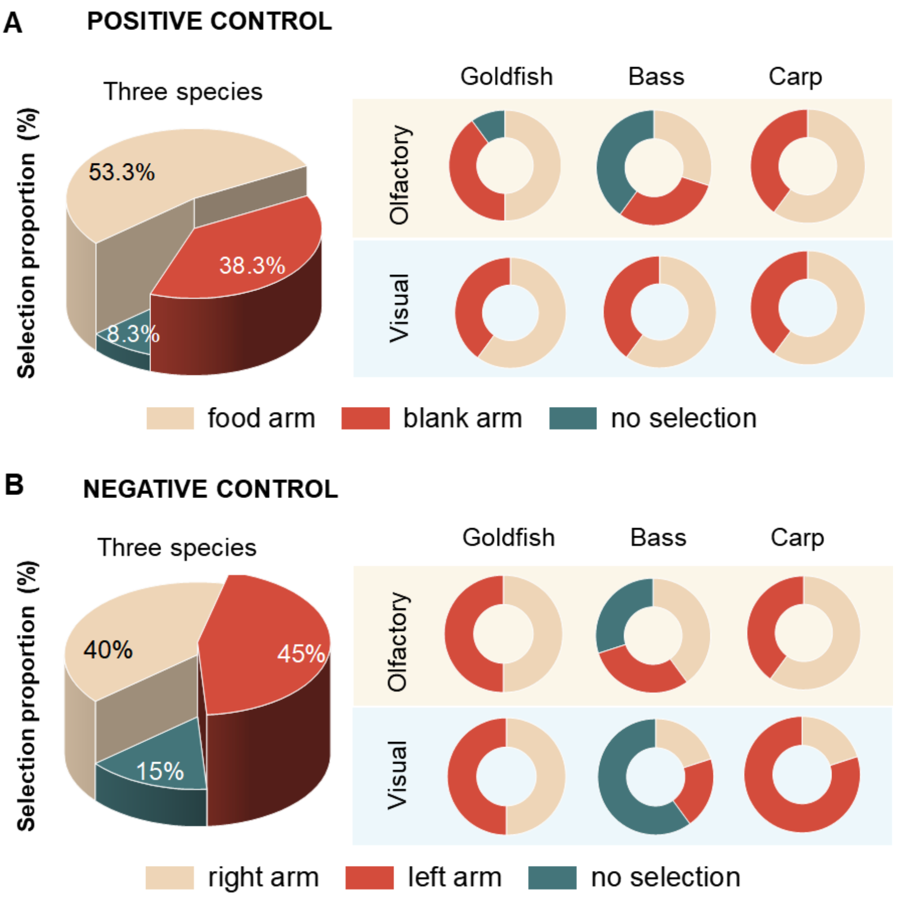


**Fig. S2. The proportion of cues selection in the control test.**

The proportion of all three species and each species that entered each cue arm for the first choice or did not make a selection. The positive control test (**A**) compared food pellets and a blank cue. The negative control test (**B**) compared blank cues in both two cue arms.

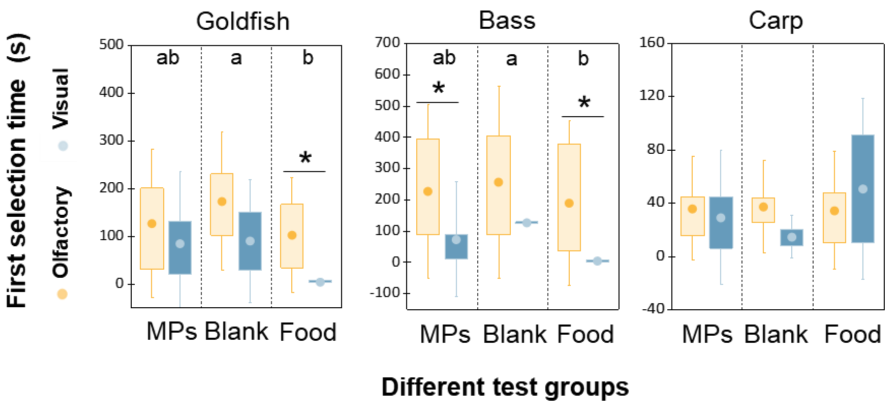


**Fig. S3. First selection time of olfactory and visual tests in different groups.**

The box represents the upper and lower quartiles of data. The solid dot represents the mean value and the whisker represents the SD value. * indicate the significant difference at the 0.05 level based on the Mann-Whitney U test. **a** and **b** indicate the significant difference at the 0.05 level based on the Kruskal-Wallis test.

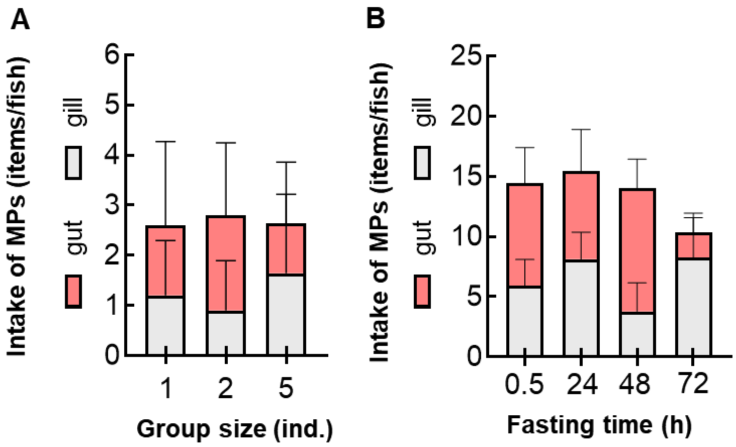


**Fig. S4. The intake of microplastic fragments by carp.**

The intake of microplastics in carp guts and gills in different group sizes (**A**) and fasting times (**B**). The bar represents the mean value of the data. The whisker represents the SD value.

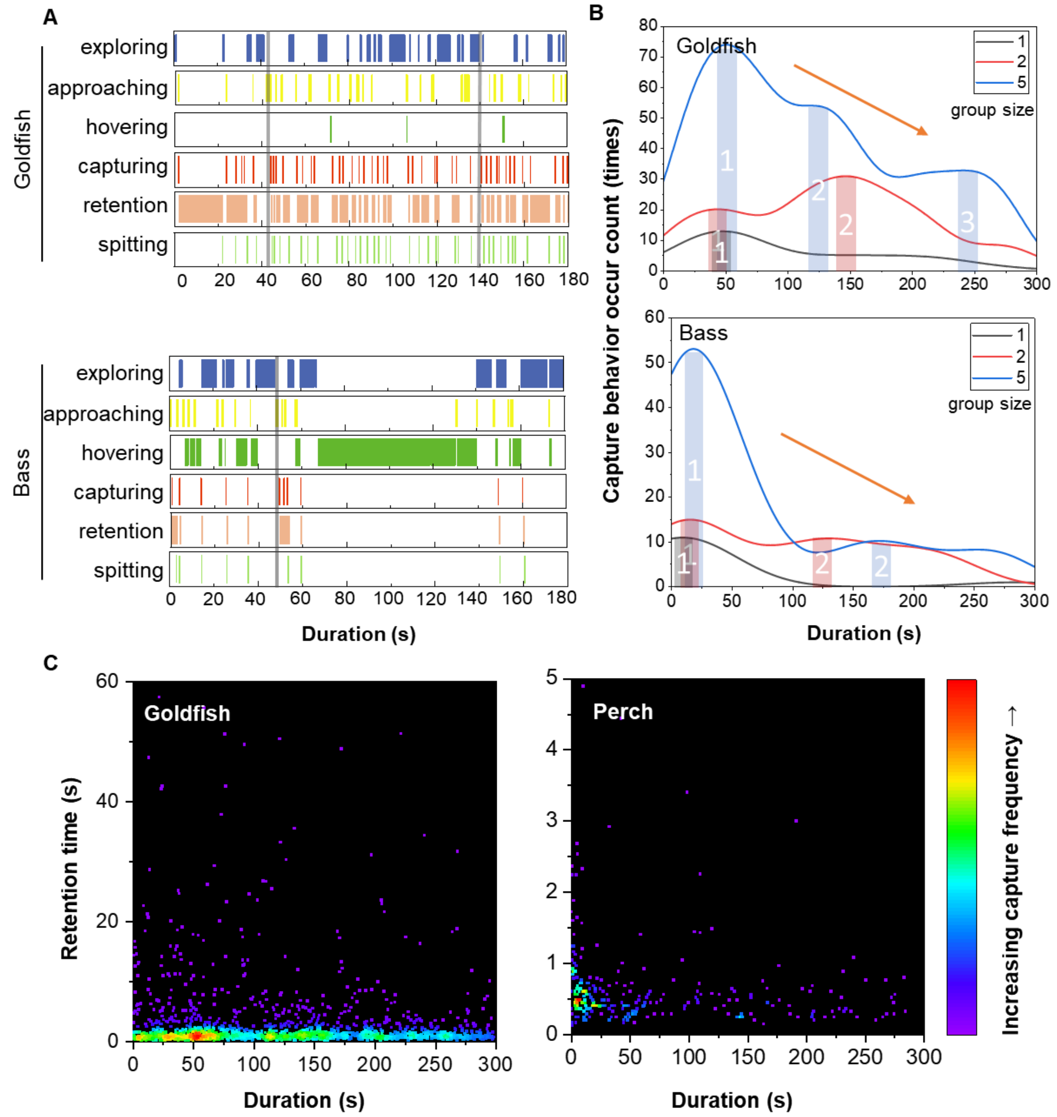


**Fig. S5. Behavior pattern of goldfish and bass to microplastic pellets.**

Sampled ethograms of one fish to microplastics in the group 3-24 (**A**). The grey line indicates the observed chasing, biting, and ramming at that moment. The distribution of capture behavior of fish during the first 5 mins in the group size test (**B**). Numbers **1**, **2**, and **3** in the sallow represent capture round 1, round 2, and round 3. A capture round means fish had the greatest number of times to capture MPs during this period. Temporal variation in retention time of goldfish and bass (**C**). Each spot represents a capture. The location of the spot represents this capture of MPs occurring at the exact time (*x*-axis) for retention (*y*-axis). The color of the spots represents the frequency of captures occurring.

## Supporting Tables

Table S1. The average total body length, weight, and mouth size of three tested fish species.

| Species | Total length (mm) | Weight (g) | Mouth size (mm) |
|---|---|---|---|
| Goldfish | 64.26 ± 4.53 | 2.51 ± 0.35 | 10.11 ± 2.10 |
| Bass | 63.64 ± 3.49 | 2.06 ± 0.31 | 13.36 ± 0.43 |
| Carp | 75.67 ± 6.59 | 2.18 ± 0.51 | 15.08 ± 1.33 |

Table S2. Hardness value and surface texture of four microplastics and food pellets.

| **Pellets types** | **Hardness (HA)** | **Surface texture** |
|---|---|---|
| PET | 8.2 ± 3.7[a] | brushed |
| PP | 81.0 ± 2.5[b] | smooth |
| PS2 | 34.4±1.4[d] | lumpy |
| PS1 | 63.4±1.1[c] | sharp |
| Food | 53.0±2.3[e] | little lumpy |
| Food in water | 9.6±1.0[a] | lumpy |

Hardness values (n=5) were presented as mean ± standard deviation (SD). The different letters after the value indicate the significant difference at the 0.05 level based on the Kruskal-Wallis test. Surface morphologies were described based on the SEM images of each particle (Fig.2).

Table S3. Definition of observed foraging behaviors of goldfish and bass to microplastics.

| **Observed behavior** | **Description** |
|---|---|
| Exploring | Random swimming in the tank |
| Approaching | The forward swimming to microplastics before capturing, often after a rapid turning |
| Hovering | Stay in front of the microplastics in the water column before capturing |
| Capturing | The quickly forward swimming and catch microplastics with mouths or the latter appears alone |
| Retention | Retaining microplastics in the mouth after capturing |
| Spitting | Expelling or ejecting microplastics from the mouth |

Table S4. Results of generalized linear mixed models examining factors affecting response time, capture frequency, and retention time of goldfish and bass, as well as the intake of MPs by carp.

| **Variable responses** | *Predictors* | *Estimates* | *CI* | *p* | *Marginal $R^2$ / Conditional $R^2$* |
|---|---|---|---|---|---|
| **response time** | **(Intercept)** | **1.58** | **1.12 – 2.22** | **0.009** | 0.019 / 0.184 |
| | group size | 1.08 | 0.98 – 1.18 | 0.124 | |
| | fasting time | 1 | 0.99 – 1.01 | 0.951 | |
| | species [goldfish] | 1.01 | 0.80 – 1.26 | 0.965 | |
| **capture frequency** | **(Intercept)** | **1.56** | **0.77 – 3.18** | **0.217** | 0.472 / 0.734 |
| | group size | 1.12 | 0.94 – 1.33 | 0.211 | |
| | fasting time | 1.01 | 1.00 – 1.02 | 0.203 | |
| | species [goldfish] | 5.19 | 3.33 – 8.07 | **<0.001** | |
| **retention time** | **(Intercept)** | **4.83** | **3.33 – 7.02** | **<0.001** | 0.077 / 0.160 |
| | group size | 1.08 | 1.00 – 1.17 | **0.049** | |
| | fasting time | 0.99 | 0.98 – 1.00 | **<0.001** | |
| | fish species [goldfish] | 0.36 | 0.28 – 0.46 | **<0.001** | |
| **intake** | **(Intercept)** | **2.12** | **0.34 – 13.15** | **0.419** | 0.029 / 0.766 |
| | group size | 1.09 | 0.64 – 1.85 | 0.747 | |
| | fasting time | 1.01 | 0.98 – 1.03 | 0.664 | |
| | site [gut] | 1.05 | 0.90 – 1.23 | 0.537 | |
